# Does parental drinking influence children's drinking? A systematic review of prospective cohort studies

**DOI:** 10.1111/add.13097

**Published:** 2015-10-16

**Authors:** Ingeborg Rossow, Patrick Keating, Lambert Felix, Jim McCambridge

**Affiliations:** ^1^ Norwegian Institute for Alcohol and Drug ResearchOsloNorway; ^2^ Faculty of Public Health and PolicyLondon School of Hygiene and Tropical MedicineLondonUK; ^3^ Faculty of Public Health and PolicyLondon School of Hygiene and Tropical MedicineLondonUK; ^4^ Department of Health SciencesUniversity of YorkYorkUK

**Keywords:** Alcohol, causal association, offspring drinking, parental drinking, prospective studies, systematic review

## Abstract

**Aims:**

To evaluate evidence of the capacity for causal inference in studies of associations between parental and offspring alcohol consumption in the general population.

**Methods:**

A systematic search for, and narrative analysis of, prospective cohort studies of the consequences of drinking, except where assessed prenatally only, or with clinically derived instruments. Primary outcome measures were alcohol use or related problems in offspring, which were collected at least 3 years after exposure measures of parental drinking. The systematic review included 21 studies comprising 26 354 families or parent–child dyads with quantitative effect measures available for each study. Criteria for capacity of causal inference included (1) theory‐driven approach and analysis; (2) analytical rigour; and (3) minimization of sources of bias.

**Results:**

Four of the 21 included studies filled several, but not all, criteria and were assessed to have some capacity for causal inference. These four studies found some evidence that parental drinking predicted drinking behaviour in adolescent offspring. The remaining 17 studies had little or no such capacity.

**Conclusions:**

There is a fairly large and consistent literature demonstrating that more parental drinking is associated with more drinking in offspring. Despite this, existing evidence is insufficient to warrant causal inferences at this stage.

## Introduction

Alcohol consumption is one of the major risk factors for loss of healthy years of life globally 1, and in high‐income countries it accounts for approximately 19% of disability‐adjusted life years (DALYs) and 27% of premature deaths among young people 2. Assessment of modifiable risk factors for young people's alcohol consumption and related harms is therefore important. In recent years the scientific and political interest in alcohol's ‘harm to others’ has grown 3, 4, 5, 6, 7, 8, including the possible harms to children from parental drinking. Numerous studies have examined both the possible effects of prenatal alcohol exposure 9, 10 and the possible effects on children living with ‘alcoholics’ or parents with serious and long‐term alcohol problems 11, 12, 13. However, less is known about how children may be affected by more normative patterns of alcohol consumption and related problems, short of those reaching clinically significant levels, including drinking at lower risk levels and heavy episodic or binge drinking. Previous reviews have addressed associations between parental and offspring drinking behaviour 14, 15 and related topics, such as parental supply of alcohol to children 16, 17. Statistically significant associations are very often observed and in many instances they are also interpreted as representing causal effects 14. However, data may be complex, and associations subject to sources of bias and confounding which may not be measured and controlled. Therefore, careful investigations of the validity of such causal inferences are needed, including thorough assessments of the extent to which other explanations for observed associations can be discounted.

Systematic reviews of prospective cohort studies offer the highest quality observational evidence available for assessment of the true consequences of parental drinking for the onset and development of alcohol use and related problems in young people. Cohort studies have the capacity to ascertain the time order of exposure and outcome and thus to rule out reverse causality. However, drawing causal inferences from observational epidemiological studies should also be based on testing theory‐driven causal hypotheses, applying sufficient analytical rigour and identification and control of sources of bias 18. The latter includes study design issues such as subject selection and retention, information acquisition and prevention of uncontrolled confounding [19. In this study we aim to review whether and to what extent prospective cohort studies in the general population provide evidence with capacity for drawing causal inferences on the true effects of parental drinking on their children's involvement with alcohol.

The importance of assessing possible causal effects of parental drinking pertains not only to a better understanding of complex mechanisms underlying young people's drinking behaviour, but it has also policy implications. Within a ‘harms to others’ framework, we are interested in the consequences of parental drinking that can be prevented by interventions which reduce parental drinking. In this perspective, both environmental influence and genetic disposition and their interaction are of interest. The literature on familial transmission of alcohol use and of alcohol use disorders (AUD) suggests several mechanisms that may explain observed associations between parental and offspring alcohol use or AUD 20. These include social learning/modelling effects; parental supply and other forms of physical access to alcohol at home; the mediating role of parenting behaviour; and activation of temperamental predispositions in the presence of environmental stress, the latter being an example of gene × environment interaction 20. A recent scoping review, which mapped the wider literature 21, identified 99 cohort studies of parental drinking and adverse outcomes in children, and 75 of these analysed drinking behaviour as an outcome. Building on this scoping review, here we review cohort studies of parental and offspring alcohol use in order to: (1) provide an overview of prospective cohort studies estimating parent–offspring drinking associations; (2) assess to what extent these studies have capacity for causal inferences; and (3) examine the strength of the evidence on the size, timing, specificity and probable mechanisms of the effects.

## Methods

### Search strategy and selection criteria

A recent scoping review of cohort studies of parental drinking and adverse outcomes in children 21 provided the basis for more stringent identification of a subset of studies concerned directly with our research questions. The search strategy and selection criteria for this scoping review are described briefly as follows: we searched five electronic databases: MEDLINE; EMBASE; PsycINFO; Global Health; and Web of knowledge, with the last searches being undertaken on 16 October 2013. One author (P.K.) performed both backward and forward searches to identify any studies that we might have missed 22. For backward searching we checked the bibliographies of included studies, while for forward searching we used Google Scholar and the Science Citation Index to identify subsequent citations of the included studies. We contacted six experts with a view to identifying additional studies. The database search strategy was devised to include terms across parental alcohol use, children and study design domains.

We sought studies that followed prospectively families or individuals of interest over a period of time, having at least two data collection points. Exposure data collection was required to precede outcome data collection in time. We included studies published in English language peer‐reviewed journals from 1980 onwards. Participants included both parents and children from general population samples; those from ‘special populations’ who may have distinct exposure–outcome relationships, e.g. mental health patients, were excluded. We excluded studies where parental drinking was measured with clinical instruments (ICD/DSM) or by brief screening tools derived from diagnostic instruments designed to identify alcohol dependence or ‘alcoholics’ [e.g. ‘The Michigan Alcoholism Screening Test (MAST)]. Clinical measures were permitted as outcomes. Studies which assessed only alcohol consumption in parents, or consumption plus problems, were included without any lower consumption limits, as were problem measures not derived from ICD/DSM criteria as they were judged probably a priori to assess less severe forms of problems. Studies in which the only parental alcohol data were maternal alcohol use measured during pregnancy were excluded.

**Figure 1:**
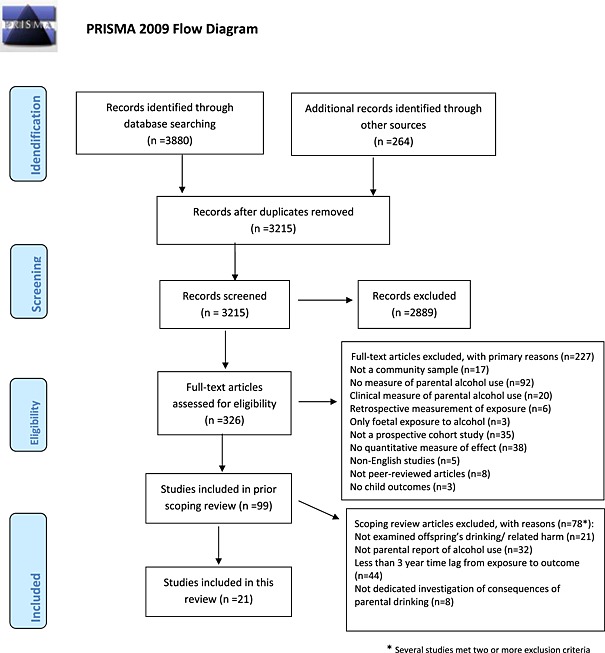
Flow diagram of study selection process

A summary of the data collection process is illustrated in the PRISMA (Preferred Reporting Items for Systematic Reviews and Meta‐Analyses) flow‐chart (Fig. 1). We followed PRISMA guidance on reporting (Supporting information, Appendix S1) and did not publish a protocol for this study, or include it in a registry. Any form of alcohol outcomes for children were included in this study, and could be assessed at any point in time, including in adulthood. We required a quantitative measure of the size of the effect of parental alcohol use on alcohol outcomes in children, such as odds ratios for binary outcomes or regression or correlation coefficients for outcomes measured on a continuous scale. We also selected studies for this review to include only those that collected exposure data from one or both of the parents, including biological or non‐biological parents, as parental reports may be more reliable than offspring's reports. Indeed, the two correlate, but offspring perceptions underestimate parental drinking 23, 24, 25, 26. We required studies to have a minimum of 3 years between data collection on exposure and outcome, as we wanted to capture enduring effects 27. Finally, we included only those studies that offered a dedicated investigation of the consequences of parental drinking (i.e. not merely inclusion of such a measure as a covariate) and which applied multivariate statistical analyses. Thus, a total of 21 studies were included (Fig. 1). These studies comprised a total of 26 354 families or parent–child dyads.

### Quality criteria and data analysis

In the assessment of these 21 studies, we built on contemporary thinking about causal inference in observational studies 18, 19, 28. We designated studies as having stronger capacity for causal inference in relation to the aims of this review if the studies had the following characteristics: (1) theory‐driven approach and analysis, including suggested mechanisms of effects, and identification of important confounding factors; (2) analytical rigour including adequate analyses to assess suggested mechanism(s), assessment of possible interactions between maternal and paternal drinking, and taking account of probable confounding factors by extent of adjustments in multivariate models; and (3) minimization of sources of bias, including having data on both parents' drinking and collected separately, exposure data collected at ages at which it could plausibly influence offspring drinking (i.e. in childhood or adolescence); a graded exposure measure in order to obtain an indication of a dose–response relationship; and sufficient statistical power to reduce Type II error risk. Regarding the theory‐driven approach, we assumed that if there is a causal effect of parental drinking on that of their children, it is likely that both parents' drinking behaviour are relevant. Therefore, we considered both parents' drinking behaviour and their additive or interactive effects to be of interest. These would preferably be self‐reported separately, and modelled to obtain additive/interactive effects. Presence of the theory‐driven approach, including suggested mechanisms and identification of important confounders, is a logical prerequisite for analytical rigour. Therefore, adjustment for a larger number of variables (e.g. maternal smoking) in the analyses does not necessarily imply better control for important confounding factors. Finally, in sensitivity analyses we assessed whether or to what extent our inclusion criteria for this review affected the main results. We summarized the outcomes of studies in the scoping review that would meet other candidate inclusion criteria for this study (e.g. having a less than 3‐year gap between exposure and outcome, or child report of parental drinking) and compared these data to the outcomes of the 21 selected studies.

## Results

The studies were conducted in six different countries: the United States (*n* = 11) [29, 30, 31, 32, 33, 34, 35, 36, 37, 38, 39; Australia (*n* = 3) [40, 41, 42, the Netherlands (*n* = 3) 43, 44, 45; New Zealand (*n* = 2) 46, 47; Finland (*n* = 1) [48; and the United Kingdom (*n* = 1)^49^. Multiple study reports were based on the same cohorts; altogether 16 distinct cohorts were identified. For each of the 21 studies, in Table 1 we have presented the study characteristics for cohort type, sample size including attrition, exposure and outcome measures and main findings, and assessed capacity for causal inference in Table 2.

**Table 1:** Overview of studies with study characteristics.

*Study*	*Characteristics*		*Exposure measure*					*Outcome(s) measure*		*Findings*	
First author, year, reference	Sample type and size	Follow‐up rate (%)	Type	Time‐frame	Categories (*n*)	By whom	Child's age	Type	Child's age(s)	Main findings, estimates, statistical significance	Adjusted for covariates
Alati, 2005 40	Birth cohort, *n* = 2386	35^a^	Drinking frequency	None	4	Only mother	At age 14	Alcohol abuse/ dependence	At age 21	Maternal daily drinking predicted alcohol problems risk for males only, OR = 2.04, *P* = 0.017	Yes
Alati, 2008 41	Birth cohort, *n* = 4363	60^b^	Usual quantity 3+	None	2	Only mother	Before/ during pregnancy/at age 5	Alcohol use frequency/ quantity	At age 14	Mothers' drinking usual 3+ before, during, and after pregnancy predicted child's greater alcohol use (various estimates for various combinations)	Yes
Alati, 2014 42	Birth cohort, *n* = 751	53^b^	Drinking categories	None	5	Both parents separate	At age 13.5	Drinking trajectories	At ages 13.5, 15.5 and 17.5	Increased maternal (OR = 2.77, *P* < 0.001) and paternal (OR = 1.40, *P* = 0.020) drinking at 13.5 years predicted a higher trajectory group. These associations did not differ for boys and girls	Yes
Armstrong, 2013 29	Community sample, *n* = 374	66^b^	Usual quantity	None	8	Both parents combined	Across ages 4.5 and 8	Alcohol use trajectories	At ages 14–17	Parental alcohol use predicted increased initial drinking (β = 0.25, *P* < 0.01), effect persisted over time	Yes
Bailey, 2006 30	Community sample, *n* = 208	55^b^	Binge drinking (5+) frequency	None	3	Both parents combined	At ages 13–14	Binge drinking (5+) frequency	At ages 15–18, 21–24 and 27	Parental 5+ drinking predicted offspring 5+ drinking at 21–24 (χ^2^ = 5.64; *P* < 0.05), but not at 15–18 and 27	No
Burk, 2011 31	Community sample, *n* = 362	67^b^	Quantity per day	None	17 cat	Both parents combined	At ages 4 and 8–9	Usual number of drinks/ occasion	In grade 10	No association between parental drinking and adolescent drinking	Yes
Casswell, 2002 46	Birth cohort, *n* = 714	77^b^	Drinking frequency and quantity	None	2 cat	Both parents separate	At age 9	Typical amount and frequency of drinking trajectories	At ages 18–26	Drinking frequency trajectories associated with mother's drinking frequency only; for men (OR = 1.7, *P* = 0.023) and women (OR = 1.8, *P* = 0.023)	Yes
Cortes, 2009 32	School students, *n* = 792	58^b^	Alcohol use, not specified	Past 30 days		Mother only	At ages 8–12	Alcohol use frequency growth	At ages 13–17	Maternal alcohol use predicted growth in child alcohol use (β = 0.10, *P* < 0.05)	Yes
Donovan, 2011 33	Community sample, *n* = 393	82	Drinking frequency	Past 6 months		Both parents combined	At age 10	Age at drinking initiation, early onset	Before age 15	Average parental drinking frequency predicted early onset of drinking (OR = 1.007, *P* < 0.05)	Yes
Duncan, 2011 34	Community sample, *n* = 256	70	Drinking frequency	None	9	Both parents combined	Ages 13, 15	Drinking frequency	Ages 18, 20	More frequent parental drinking predicted increased youth drinking over time (β = 0.10, *P* < 0.05)	Yes
Fergusson, 1995 47	Birth cohort, *n* = 953	75^b^	Typical weekly volume	None		Both parents combined	Age 11	Amount/ occasion, hazardous drinking	Ages 14, 16	Parental drinking did not predict drinking at age 14, and was not directly associated with hazardous drinking at age 16	No (at 14) and yes (at 16)
Guo, 2001 35	School students, *n* = 808	72^b^	Alcohol use, not specified	None		Both parents combined	Ages 10, 14, 16	Alcohol abuse/ dependence	Age 21	Only parental alcohol use at age 16 predicted alcohol abuse (OR = 1.42, *P* < 0.01) and alcohol dependence (OR = 1.65, *P* < 0.01)	Yes
Hawkins, 1997 36	Students, *n* = 757	72^b^	Drinking frequency	None		Both parents combined	Ages 10–11	Alcohol initiation, alcohol misuse	Ages 17–18	Parental drinking predicted earlier drinking initiation (β = –0.19, *P* < 0.05), no direct association with alcohol misuse at 17–18	Yes
Latendresse, 2008 48	Cohort twins, *n* = 4731	58^b^	Drinking frequency, intoxication frq	Current	9	Both parents combined	Ages 11–12	Drinking behaviour	Ages 14 and 17.5	Parental drinking behaviours predicted child's alcohol use and intoxication at ages 14 and 17 (8 path coefficients, range 0.02–0.16, *P* < 0.001 for all)	Yes
Macleod, 2008 49	Birth cohort, *n* = 4064	27^b^	Parental drinking,	None	3 cat,	Both parents separate	Ages 0–4	Alcohol use	Age 10	Maternal drinking predicted only alcohol use (OR = 2.6, *P* < 0.01) , no association with paternal drinking	Yes
Mares, 2011 43	Families, *n* = 428	76	Drinking frequency, volume	Past 4 weeks	6 (frequency)	Both parents separate	Ages 13–16	Excessive alcohol use, related problems	Ages 17–20	Paternal, but not maternal, drinking predicted only excessive drinking (β = 0.16 for older and β = 0.17 for younger adolescents, *P* < 0.05)	Yes
Pears, 2007 37	103 families	68^b^	Drinking frequency	None		Both parents combined	Ages 9–10	Alcohol use frequency	Ages 16–18	Grandparents' alcohol use predicted parents' alcohol use (path coefficient = 0.22, *P* < 0.05)	Yes
Poelen, 2007 44	Twin families, *n* = 1779	47^b^	Drinking frequency	None	3	Both parents separate	Ages 12–25	Regular drinking	Ages 14–27, 19–32	Only maternal drinking few times/week predicted regular drinking 7 years (OR = 1.78, *P* < 0.001) later	Yes
Poelen, 2009 45	Twin families, *n* = 1796	48^b^	Drinking frequency	None	3	Both parents separate	Ages 12–25	Problem drinking (CAGE)	Ages 19–32	Only paternal drinking a few times/week predicted problem drinking 7 years later (OR = 1.78, *P* < 0.05). This did not differ for boys and girls	Yes
Tyler, 2006 38	Youth cohort, *n* = 244	45^c^	Binge drinking (5+)	Past 30 days	2	Mother only	Ages 10–12	Binge drinking (5+)	Ages 14–16, 16–18	Mother's binge drinking predicted binge drinking at ages 14–16 (β = 0.171, *P* < 0.01), not at ages 16–18	Yes
Webster, 1989 39	Community sample, *n* = 420 families	Not clear	Volume	None		Both parents separate	Mean age 16–17	Alcohol amount per week	Mean ages 33–34 (SD 8.4–10.0)	Father's drinking (partial *r* = 015, *P* = 0.05) and mother's drinking (partial *r* = 0.16, *P* = 0.04) predicted alcohol use in sons, only father's drinking (partial *r* = 0.29, *P* < 0.001) predicted alcohol use in daughters	Yes

Sample size is the number of people in the (multivariate) analysis.

a Proportion that was followed‐up and completed Composite International Diagnostic Interview (CIDI);

b our calculation based on the figures in the article;

c net sample as proportion of initial gross sample when missing data excluded. TF = time‐frame; cat =  number of categories; parents comb = measure of parental drinking combined; CAGE = Cut‐down, Annoyed, Guilt, Eye‐opener; OR = odds ratio; SD = standard deviation.

**Table 2:** Assessments of study characteristics favourable to causal inference and evaluation of study capacity for causal inference.

*Author, year, reference*	*Study characteristics favourable to causal inference*							*Notes on outcome measure*	*Capacity for causal inference*
	*Main focus on parent–offspring drinking association*	*Theory‐driven analyses aimed at assessing causality*	*Identification of important confounding factors*	*Sample size*	*Exposure measure graded*	*Relevant period*	*Assessment of parental drinking interaction*		
Alati, 2005 40	No	No	No	Large	Yes	Yes	No		Little
Alati, 2008 41	Yes	No	No	Large	No	No, long before outcome	No		Little
Alati, 2014 42	Yes	Suggests parenting may mediate the association. Analysis not clearly aimed at addressing causality	Time‐varying covariates included	Acceptable	Yes	Yes	No	Limited data on key measure	Some
Armstrong 2013 29	No	No	No	On the smaller side	Yes	Somewhat early	No		Little
Bailey, 2006 30	No	No	No	Small	Yes	Yes	No		Little
Burk, 2011 31	No	No	No	On the smaller side	Yes	Somewhat early	No		Little
Casswell, 2002 46	No	No	No	Acceptable	No	Many years from exposure to outcome	No	Trajectories from ages 18–26	Little
Cortes, 2009 32	No	No	Unclear	Acceptable	Vaguely described	Yes	No		Little
Donovan, 2011 33	No	No	No	On the smaller side	Yes	Yes	No	Validation of outcome measure	Little
Duncan, 2011 34	No	No	No	Small	Yes	Yes	No	Crude	Little
Fergusson, 1995 47	No	No	No	Acceptable	Yes	Yes	Additive effect implied		Little
Guo, 2001 35	No	No	No	Acceptable	Not described	Yes	No		Little
Hawkins, 1997 36	No	No	No	Acceptable	Vaguely described	Yes	No	Construction and validity not clear	Little
Latendresse, 2008 48	Yes	Suggested mediation mechanisms examined	3 covariates included, probably lacks important confounding factors	Large	Yes	Yes	No	Two aspects (similar for parents and children) modelled	Some
Macleod, 2008 49	No	No	No	Large	No	Yes	No	Low‐prevalent outcome and substantial missing data	Little
Mares, 2011 43	Yes	Suggested modelling effects via alcohol communication	No	On the lower side	Yes	Yes	No		Some
Pears, 2007 37	Yes	Suggested indirect effects through parental discipline and offspring's inhibitory control	No	Very small	Vaguely described	Yes	No	Adjacent survey years combined	Some
Poelen, 2007 44	No	No	No	Large	Yes	Partly	No		Little
Poelen, 2009 45	No	No	No	Large	Yes	Partly	No		Little
Tyler, 2006 38	No	No	No	Small	No		No		Little
Webster, 1989 39	No	No	No	Small	Yes	No			Little

The exposure measure varied substantially between the studies with regard to type of drinking behaviour (e.g. drinking frequency, typical weekly volume), age of exposure and putative relationship to outcomes (from before pregnancy to young adulthood), and whose drinking behaviour was measured (only mother, only father, separate measures for both parents or combined measure for both parents; Table 1).

The outcome was one or several measures of drinking behaviour (e.g. drinking frequency, early onset of drinking or heavy episodic drinking frequency) in 16 of the studies. In five studies the outcome was some kind of alcohol‐related problem (e.g. alcohol dependence), either as a single outcome (three studies) 35, 40, 45 or in addition to a measure of drinking behaviour (two studies) [36, 43. In 13 of the studies the outcome measures were obtained only or mainly during the teenage years, whereas in seven studies the outcome measures were obtained mainly or only in young adulthood 30, 35, 39, 40, 44, 45, 46, and in one study at the age of 10 years 49. In light of observed heterogeneity and the consequent lack of data appropriate for meta‐analysis, we undertook a narrative synthesis of included study findings and risk of bias.

The vast majority (19 of 21 studies) reported at least one positive association between parental drinking and offspring's alcohol‐related outcome, while only two studies 31, 47 found no statistically significant association. This pattern held for both adolescent and young adult outcomes (Table 1). Of eight studies that examined mother's and father's possible drinking consequences separately, three studies reported that both parents' drinking behaviour predicted that of the child 33, 39, 42, three studies found that only mother's drinking predicted the outcome 44, 46, 49, and two studies found that only father's drinking predicted the outcome 43, 45 (Table 1). Among four studies addressing same sex versus opposite sex associations between parent and offspring drinking 39, 42, 45, 46, the findings were mixed (Table 1).

Next, we assessed the studies' capacity for causal inference according to the aims of this study and the evaluation framework described previously in relation to parental drinking and alcohol‐related outcomes in offspring. All studies had some favourable characteristics in this respect; for instance, graded exposure measures or large sample sizes (Table 2). However, the majority of the studies were not well designed to evaluate possible causation and lacked an explicit theoretical conceptualization of their research aims. In fact, none of the studies identified and accounted for theory‐driven important confounding factors in order to interrogate observed associations. Therefore, we found that none of the 21 studies could be considered as having strong capacity for causal inference. Four studies 37, 42, 43, 48 were found to have some inferential capacity in this respect and the remaining 17 studies had little or no such capacity (see Table 2 for a summary of the basis of categorization of each included study).

Among the four studies 37, 42, 43, 48 with some capacity for causal inference, all found some evidence that parental drinking predicted drinking behaviour in offspring (Table 3). Three of these studies had clear theory‐driven analyses of the association between parental and offspring drinking 37, 43, 48. They examined specific mediation mechanisms, assuming that the association between parental and offspring drinking was mediated by either parenting practices 48, by alcohol‐specific communication 43 or by poor inhibitory control in offspring 37. Conversely, the study by Alati and co‐workers 42 accounted for some theory‐driven covariates in the analyses, but not within a clear framework of testing causal mechanisms, thereby hampering substantive interpretation of the reported findings.

**Table 3:** Main findings in studies with some capacity for causal inference.

*First author, publication year*	*Main findings and estimates*	*Adjustment for confounding factors*
Alati, 2014 42	Increased maternal and paternal drinking (on a five‐category ordinal scale) at 13.5 years predicted a higher (compared to a lower) drinking trajectory group through ages 15.5 and 17.5. Paternal drinking: OR = 1.40, maternal drinking: OR = 2.77. These associations did not differ for boys and girls	Time‐dependent covariates of anti‐social behaviour, SES and harsh parental discipline
Latendresse, 2008 48	Parental (most probably paternal) drinking behaviour at offsprin's age 11 predicted offspring's drinking behaviour 3 and 6.5 years later (at ages 14 and 17.5). Larger total effect at 17.5 years (β = 0.222) than at age 14 (β = 0.038). As hypothesized, both effects mediated partly by parental monitoring and discipline; more so at age 14	Gender, family structure, and zygosity were included as co‐variates in multiple mediation models
Mares, 2011 43	Paternal, but not maternal, frequency of alcohol use in the past 4 weeks was associated positively with child's excessive drinking (frequency of 5+ drinks in past 4 weeks) 3 years later in direct path models of both younger and older sibling (ages 13 and 15 at T1), β = 0.16/0.17. Paternal, but not maternal, alcohol‐related problems (a sum‐score scale) were also associated with offspring excessive drinking 3 years later in direct path models of both sibling, β = 0.13/.14. In contrast to a priori hypotheses, both paternal and maternal alcohol‐related problems predicted more rather than less alcohol‐specific communication with offspring, which in turn was associated with less excessive drinking (β = –0.14) and less alcohol‐related problems (β = –0.13) in offspring in indirect path models	Only for adolescent drinking at T1
Pears, 2007 37	Parental drinking frequency (combined) at age 9–12 predicted child's drinking frequency at age 16–18 (standardized β = 0.22) in path model. This association was not, however, mediated by inhibitory control, as hypothesized	No confounding variable was identified and accounted for in the analysis

OR = odds ratio; SES = socio‐economic status.

The study by Mares and co‐workers 43 found direct effects of paternal, but not maternal drinking; however, the apparent differential effects may be due to insufficient statistical power and model misspecification (intercorrelated measures of maternal and paternal drinking were estimated simultaneously). The findings also, in part, indicated indirect effects of parental alcohol‐related problems through parental–child communication: more alcohol‐related problems in parents predicted more alcohol‐specific communication, which again predicted less excessive drinking and alcohol‐related problems in offspring. However, the estimated indirect paths did not display a consistent or easily interpretable pattern and the statistically significant indirect paths were in contrast with the direct paths, which were not statistically significant. Thus, the study did not provide clear evidence on the hypothesized mediating effect of alcohol‐specific communication. The study by Pears and co‐workers 37 did not find any mediation effect of poor inhibitory control in offspring, which may well be due to insufficient statistical power, or there may be no such effect.

The study by Latendresse and co‐workers 48 is particularly noteworthy in the context of our research aims. The authors found that the association between parental (probably mainly paternal) and offspring drinking was mediated in part by parental monitoring and discipline, and more so in early than in late adolescence. Although mediation was stronger at age 14, the effect of parental drinking was much larger at age 17. Thus this study offers more insight into a probable mechanism and its relative importance with respect to timing. Three covariates were adjusted for in the analyses, but other potentially confounding factors were not identified and accounted for, thus limiting capacity for causal inference. Moreover, it is also quite possible that if there is a causal and partly mediated effect of parental drinking it may be an additive or interaction effect of both parents' drinking behaviour, which was not addressed in this study, or in any of the other studies included in this review. Consequently, findings on the size, timing, specificity and probable mechanisms of the effects are very limited across these studies.

Finally, we assessed whether our inclusion criteria for this analytical review had impacted upon our findings. The 21 studies included in our review differed very little from the findings of 28 excluded studies with either child report of exposure, or a gap of less than 3 years between exposure and outcome measurement. A combined measure for both parents tended to be used when parental drinking was reported by offspring, and only 55% of these studies found an association between parental and offspring drinking, compared to 78% of studies using parental report (χ^2^ = 3.51, *P* = 0.06).

## Discussion

This study is the first systematic review of cohort studies which interrogates the basis for causal inference on the effects of parental drinking on children's alcohol outcomes. It has demonstrated that among the many prospective cohort studies that have addressed whether and to what extent parental drinking predicts drinking behaviour in offspring, few have been designed to measure validly the effects of parent drinking on the drinking of their offspring. Almost all prospective studies on this topic have found that parental drinking predicts drinking behaviour in their children; that is, when one or both parents drink more, their offspring are more likely to report more drinking or more alcohol‐related problems later on than others in the cohort. Findings on the relative effects of paternal versus maternal drinking are different in the studies by Alati 42 and Mares 43, with maternal drinking more important in the former and paternal drinking more so in the latter. This overall consistency in findings is, however, not sufficient by itself to indicate a causal relationship 28. The somewhat mixed findings regarding the differential impacts of maternal and paternal drinking, the sparse use of theory‐driven analyses, and thus the lack of identification and control for relevant confounding factors, the small data sets and consequent limited capacity for detecting associations of moderate magnitude, are all factors that imply caution is warranted about the consequences of parental drinking under consideration.

Closely related to the topic of this review is the literature on familial transmission of alcohol misuse. Relying on both twin and adoption studies this literature suggests that genetic predisposition and interactions between genes and environment are important 50. A striking observation, therefore, is that studies included in this review, and particularly studies using twin data 44, 45, 48, did not address these factors. Possible mechanisms that were suggested and examined in some studies in this review 43, 48, 51 were all in the behavioural domain. Thus, it seems that data from designs other than prospective cohort studies are more informed by genetic data and gene–environment interactions, and it remains to be seen how far rigorously designed systematic reviews may alter evidential claims about genetic heritability in a wider range of study designs.

The four studies with some capacity for causal inference all found that parental drinking predicted greater involvement in drinking in offspring. However, the possibility that these observed associations are spurious needs consideration. Some possible sources of spurious associations are as follows: (a) common local environment (neighbourhood, community) influences on both parental and offspring drinking, such as physical access to alcohol and price; (b) common cultural or religious factors including both those that enhance and limit or proscribe drinking that affect both parental and offspring drinking; and (c) parental comorbidity/temperament and other psychobiological factors affected by genetic transmission. These factors may either moderate or mediate mechanisms, or both, and are seldom addressed in the studies included in this review. Failure to demonstrate mediation effects as they were hypothesized in two 37, 43 of three studies 37, 43, 48, all of which were assessed to have some capacity for causal inference, and some inconsistency regarding possible effects of maternal drinking, may well be due to insufficient statistical power and model misspecification, meaning that the hypothesized mediation effects and specific effects of maternal drinking should not be discarded from further investigations.

As well as evaluation of the included studies, consideration of the strengths and limitations of this study is appropriate. A fairly large literature on cohort studies of parental and offspring drinking was identified through extensive and systematic literature searches. Applying a set of criteria for drawing causal inferences, including theoretical underpinning and analytical rigour, enhanced the systematic evaluation of the studies' contributions in this respect. This process has also been made as transparently as possible, permitting readers to assess its rigour and its limitations. Studies from different national and cultural contexts were identified, although these were restricted entirely to Anglophone and northern European countries. The selection of studies was restricted to those published in the English language, implying that relevant studies in other languages may exist, but have not been identified in this review. Diverse measures of exposures and outcomes entailed difficulties in conducting quantitative syntheses and it may have been possible to pursue quantitative investigations, notwithstanding the heterogeneity we encountered.

The lack of standardization in measurement may also be regarded as a limitation of the literature as a whole, hampering comparability across studies. For instance, with respect to adolescents' drinking behaviour, there is a distinction between sipping and consumption of full beverages 52, and relatedly it is worth considering that age of onset/initiation as an outcome measure (as in 33, 36, 49) may have a distinct relationship to parental drinking. It may also have different consequences from other alcohol involvement outcomes investigated here, which may also be heterogeneous in this regard, and the need for an intergenerational life‐course perspective should be considered 53.

The determination of study quality did not consider self‐report bias in both exposure and outcome measures, and is otherwise absent from this study design except in separating the two reports in time. Self‐reported drinking behaviour is often under‐reported, and this leads to a biased estimate of the associations with consequences 27. The possibility of publication bias needs also to be considered 54. As null‐findings are less likely to be published, the observed associations in the vast majority of studies included here may represent an exaggeration of the true picture. Due to the nature of the literature, we have not been able to assess this quantitatively. Finally, our study findings need to be interpreted within the context of the emphasis we have placed on the testing of theory‐driven causal hypotheses and other aspects of the design of this systematic review.

Strategies to prevent harmful drinking in young people and its acute and long‐term health and social consequences may target parents and parental drinking and include general population strategies 55, 56 and specific parent‐targeted programmes 57, 58, but the effectiveness of the latter is contingent upon an underlying causal effect of parental drinking on that of their children. The findings from studies with some capacity for causal inference suggest that such effects may actually exist. This study has demonstrated that there is currently little strong evidence, however, of a causal effect of parental drinking on that of their children. More well‐designed theory‐driven cohort studies addressing the possible influence of parental drinking on that of their children, as well as other putative risk factors, are needed urgently in order to understand more clearly the true burden of alcohol's harm to others, and to determine the most appropriate ways to prevent intergenerational alcohol problems.

### Declaration of interest

None.

## Supporting information

Supporting info item

## References

[1] Rehm J. , Mathers C. D. , Popova S. , Thavorncharoensap M. , Teerawattananon Y. , Patra J. Global burden of disease and injury and economic cost attributable to alcohol use and alcohol‐use disorders. Lancet 2009; 373: 2223–33. 19560604 10.1016/S0140-6736(09)60746-7

[2] Toumbourou J. W. , Stockwell T. , Neighbors C. , Marlatt G. , Sturge J. , Rehm J. Interventions to reduce harm associated with adolescent substance use. Lancet 2007; 369: 1391–401. 17448826 10.1016/S0140-6736(07)60369-9

[3] Giesbrecht N. , Cukier S. , Steeves D. A. N. Collateral damage from alcohol: implications of ‘second‐hand effects of drinking’ for populations and health priorities. Addiction 2010; 105: 1323–5. 20653610 10.1111/j.1360-0443.2009.02884.x

[4] Room R. , Ferris J. , Laslett A.‐M. , Livingston M. , Mugavin J. , Wilkinson C. The drinker's effect on the social environment: a conceptual framework for studying alcohol's harm to others. Int J Environ Res Public Health 2010; 7: 1855–71. 20617064 10.3390/ijerph7041855 PMC2872341

[5] Casswell S. , You R. Q. , Huckle T. Alcohol's harm to others: reduced wellbeing and health status for those with heavy drinkers in their lives. Addiction 2011; 106: 1087–94. 21226881 10.1111/j.1360-0443.2011.03361.x

[6] Laslett A.‐M. , Catalano P. , Chikritzhs Y. , Dale C. , Doran C. , Ferris J. *et al.* The Range and Magnitude of Alcohol's Harm to Others. AER Centre for ALcohol Policy Research, Turning Point Alcohol and Drug Centre: Fitzroy, Victoria; 2010.

[7] Babor T. F. Commentary on Laslett *et al*. (2011): Alcohol‐related collateral damage and the broader issue of alcohol's social costs. Addiction 2011; 106: 1612–3. 21815923 10.1111/j.1360-0443.2011.03578.x

[8] Greenfield T. K. , Ye Y. , Kerr W. , Bond J. , Rehm J. , Giesbrecht N. Externalities from alcohol consumption in the 2005 US National Alcohol Survey: implications for policy. Int J Environ Res Public Health 2009; 6: 3205–24. 20049257 10.3390/ijerph6123205 PMC2800345

[9] Henderson J. , Kesmodel U. , Gray R. Systematic review of the fetal effects of prenatal binge‐drinking. J Epidemiol Community Health 2007; 61: 1069–73. 18000129 10.1136/jech.2006.054213 PMC2465662

[10] Riley E. P. , Infante M. A. , Warren K. R. Fetal alcohol spectrum disorders: an overview. Neuropsychol Rev 2011; 21: 73–80. 21499711 10.1007/s11065-011-9166-x PMC3779274

[11] Johnson J. L. , Leff M. Children of substance abusers: overview of research findings. Pediatrics 1999; 103: 1085–99. 10224196

[12] Manning V. , Best D. W. , Faulkner N. , Titherington E. New estimates of the number of children living with substance misusing parents: results from UK national household surveys. BMC Public Health 2009; 9: 377. 19814787 10.1186/1471-2458-9-377 PMC2762991

[13] Seilhamer R. A. , Jacob T. Family factors and adjustment of children of alcoholics In: Windle M., Searles J. S., editors. Children of Alcoholics Critical Perspectives. New York: Guilford Press; 1990, pp. 168–86.

[14] Ryan S. M. , Jorm A. F. , Lubman D. I. Parenting factors associated with reduced adolescent alcohol use: a systematic review of longitudinal studies. Aust NZ J Psychiatry 2010; 44: 774–83. 10.1080/00048674.2010.501759 20815663

[15] Hayes L. , Smart D. , Toumbourou J. W. , Sanson A. Parenting Influences on Adolescent Alcohol Use. Canberra: Australian Institute of Family Studies; 2004.

[16] Gilligan C. , Kypri K. , Johnson N. , Lynagh M. , Love S. Parental supply of alcohol and adolescent risky drinking. Drug Alcohol Rev 2012; 31: 754–62. 22340514 10.1111/j.1465-3362.2012.00418.x

[17] Kaynak Ö. , Winters K. C. , Cacciola J. , Kirby K. C. , Arria A. M. Providing alcohol for underage youth: what messages should we be sending parents? J Stud Alcohol Drugs 2014; 75: 590–605. 24988258 10.15288/jsad.2014.75.590 PMC4108600

[18] Pearl J. Causal inference in statistics: an overview. Stat Surv 2009; 3: 96–146.

[19] Rothman K. , Greenland S. Causation and causal inference in epidemiology. Am J Public Health 2005; 95: S144–50. 16030331 10.2105/AJPH.2004.059204

[20] Leonard K. E. , Eiden R. D. Marital and family processes in teh context of alcohol use and alcohol disorders. Annu Rev Clin Psychol 2007; 3: 285–310. 17716057 10.1146/annurev.clinpsy.3.022806.091424 PMC2667243

[21] Rossow I. , Felix L. , Keating P. , McCambridge J. Parental drinking and adverse outcomes in children—a scoping review of cohort studies. Drug Alcohol Rev 2015; doi: 10.1111/dar.12319. 10.1111/dar.12319 PMC4950034 26332090

[22] Greenhalgh T. , Peacock R. Effectiveness and efficiency of search methods in systematic reviews of complex evidence: audit of primary sources. BMJ 2005; 331: 1064–5. 16230312 10.1136/bmj.38636.593461.68 PMC1283190

[23] Ary D. V. , Tildesley E. , Hops H. , Andrews J. A. The influence of parent, sibling, and peer modeling and attitudes on adolescent use of alcohol. Subst Use Misuse 1993; 28: 853–80. 10.3109/10826089309039661 8359945

[24] White H. R. , Johnson V. , Buyske S. Parental modeling and parenting behavior effects on offspring alcohol and cigarette use: a growth curve analysis. J Subst Abuse 2000; 12: 287–310. 11367605 10.1016/s0899-3289(00)00056-0

[25] Aas H. , Jakobsen R. , Anderssen N. Predicting 13‐year‐olds' drinking using parents' self‐reported alcohol use and restrictiveness compared with offspring's perception. Scand J Psychol 1996; 37: 113–20. 8711450 10.1111/j.1467-9450.1996.tb00644.x

[26] Dielman T. E. , Leech S. L. , Loveland‐Cherry C. Parents' and children's reports of parenting practices and parent and child alcohol use. Drugs Soc 1995; 8: 83–101.

[27] McCambridge J. , McAlaney J. , Rowe R. Adult consequences of late adolescent alcohol consumption: a systematic review of cohort studies. PLOS Med 2011; 8e1000413. 10.1371/journal.pmed.1000413 PMC3035611 21346802

[28] Rothman K. J. , Greenland S. , Poole C. , Lash T. L. Causation and causal inference In: Rothman K. J., Greenland S., Lash T. L., editors. Modern Epidemiology, 3rd edn. Philadelphia, PA: Lippincott, Williams & Wilkins; 2008 p. 5–31.

[29] Armstrong J. M. , Ruttle P. L. , Burk L. R. , Costanzo P. R. , Strauman T. J. , Essex M. J. Early risk factors for alcohol use across high school and its covariation with deviant friends. J Stud Alcohol Drugs 2013; 74: 746. 23948534 10.15288/jsad.2013.74.746 PMC3749318

[30] Bailey J. A. , Hill K. G. , Oesterle S. , Hawkins J. D. Linking substance use and problem behavior across three generations. J Abnorm Child Psychol 2006; 34: 263–82. 16752101 10.1007/s10802-006-9033-z

[31] Burk L. R. , Armstrong J. M. , Goldsmith H. H. , Klein M. H. , Strauman T. J. , Costanzo P. R. *et al.* Sex, temperament, and family context: how the interaction of early factors differentially predict adolescent alcohol use and are mediated by proximal adolescent factors. Psychol Addict Behav 2011; 25: 1. 21443307 10.1037/a0022349 PMC3174803

[32] Cortes R. C. , Fleming C. B. , Mason W. A. , Catalano R. F. Risk factors linking maternal depressed mood to growth in adolescent substance use. J Emot Behav Disord 2009; 17: 49–64. 20160836 10.1177/1063426608321690 PMC2493414

[33] Donovan J. E. , Molina B. S. G. Childhood risk factors for early‐onset drinking. J Stud Alcohol Drugs 2011; 72: 741. 21906502 10.15288/jsad.2011.72.741 PMC3174021

[34] Duncan S. C. , Gau J. M. , Duncan T. E. , Strycker L. A. Development and correlates of alcohol use from ages 13‐20. J Drug Educ 2011; 41: 235–52. 22125920 10.2190/DE.41.3.a PMC3597217

[35] Guo J. , Hawkins J. D. , Hill K. G. , Abbott R. D. Childhood and adolescent predictors of alcohol abuse and dependence in young adulthood. J Stud Alcohol 2001; 62: 754. 11838912 10.15288/jsa.2001.62.754 PMC1868672

[36] Hawkins J. D. , Graham J. W. , Maguin E. , Abbott R. D. , Hill K. G. , Catalano R. F. Exploring the effects of age of alcohol use initiation and psychosocial risk factors on subsequent alcohol misuse. J Stud Alcohol 1997; 58: 280. 9130220 10.15288/jsa.1997.58.280 PMC1894758

[37] Pears K. , Capaldi D. M. , Owen L. D. Substance use risk across three generations: the roles of parent discipline practices and inhibitory control. Psychol Addict Behav 2007; 21: 373. 17874888 10.1037/0893-164X.21.3.373 PMC1988842

[38] Tyler K. A. , Stone R. T. , Bersani B. Examining the changing influence of predictors on adolescent alcohol misuse. J Child Adoles Subst Abuse 2006; 16: 95–114.

[39] Webster D. W. , Harburg E. , Gleiberman L. , Schork A. , DiFranceisco W. Familial transmission of alcohol use: I. Parent and adult offspring alcohol use over 17 years—Tecumseh, Michigan. J Stud Alcohol Drugs 1989; 50: 557. 10.15288/jsa.1989.50.557 2586109

[40] Alati R. , Najman J. M. , Kinner S. A. , Mamun A. A. , Williams G. M. , O'Callaghan M. *et al.* Early predictors of adult drinking: a birth cohort study. Am J Epidemiol 2005; 162: 1098–107. 16236998 10.1093/aje/kwi320

[41] Alati R. , Clavarino A. , Najman J. M. , O'Callaghan M. , Bor W. , Mamun A. A. *et al.* The developmental origin of adolescent alcohol use: findings from the Mater University Study of Pregnancy and its outcomes. Drug Alcohol Depend 2008; 98: 136–43. 18639392 10.1016/j.drugalcdep.2008.05.011

[42] Alati R. , Baker P. , Betts K. , Connor J. , Little K. , Sanson A. *et al.* The role of parental alcohol use, parental discipline and antisocial behaviour on adolescent drinking trajectories. Drug Alcohol Depend 2014; 134: 178–84. 24479151 10.1016/j.drugalcdep.2013.09.030

[43] Mares S. H. W. , van der Vorst H. , Engels R. C. M. E. , Lichtwarck‐Aschoff A. Parental alcohol use, alcohol‐related problems, and alcohol‐specific attitudes, alcohol‐specific communication, and adolescent excessive alcohol use and alcohol‐related problems: an indirect path model. Addict Behav 2011; 36: 209–16. 21084165 10.1016/j.addbeh.2010.10.013

[44] Poelen E. A. P. , Scholte R. H. J. , Willemsen G. , Boomsma D. I. , Engels R. C. M. E. Drinking by parents, siblings, and friends as predictors of regular alcohol use in adolescents and young adults: a longitudinal twin‐family study. Alcohol Alcohol 2007; 42: 362–9. 17537828 10.1093/alcalc/agm042

[45] Poelen E. A. P. , Engels R. C. M. E. , Scholte R. H. J. , Boomsma D. I. , Willemsen G. Predictors of problem drinking in adolescence and young adulthood. Eur Child Adolesc Psychiatry 2009; 18: 345–52. 19205784 10.1007/s00787-009-0736-x

[46] Casswell S. , Pledger M. , Pratap S. Trajectories of drinking from 18 to 26 years: identification and prediction. Addiction 2002; 97: 1427–37. 12410783 10.1046/j.1360-0443.2002.00220.x

[47] Fergusson D. M. , Horwood L. J. , Lynskey M. T. The prevalence and risk factors associated with abusive or hazardous alcohol consumption in 16‐year‐olds. Addiction 1995; 90: 935–46. 7663315 10.1046/j.1360-0443.1995.9079356.x

[48] Latendresse S. J. , Rose R. J. , Viken R. J. , Pulkkinen L. , Kaprio J. , Dick D. M. Parenting mechanisms in links between parents' and adolescents' alcohol use behaviors. Alcohol Clin Exp Res 2008; 32: 322–30. 18162066 10.1111/j.1530-0277.2007.00583.x PMC2504716

[49] Macleod J. , Hickman M. , Bowen E. , Alati R. , Tilling K. , Smith G. D. Parental drug use, early adversities, later childhood problems and children's use of tobacco and alcohol at age 10: birth cohort study. Addiction 2008; 103: 1731–43. 18705686 10.1111/j.1360-0443.2008.02301.x

[50] Agrawal A. , Lynskey M. T. Are there genetic influences on addiction: evidence from family, adoption and twin studies. Addiction 2008; 103: 1069–81. 18494843 10.1111/j.1360-0443.2008.02213.x

[51] Coggan C. , Patterson P. , Brewin M. , Hooper R. , Robinson E. Evaluation of the Waitakere Community Injury Prevention Project. Inj Prev 2000; 6: 130–4. 10875670 10.1136/ip.6.2.130 PMC1730604

[52] Wadolowski M. , Bruno R. , Aiken A. , Stone C. , Najman J. , Kypri K. *et al.* Sipping, drinking, and early adolescent alcohol consumption: a cautionary note. Alcohol Clin Exp Res 2015; 39: 350–4. 25684054 10.1111/acer.12613

[53] Maimaris W. , McCambridge J. Age of first drinking and adult alcohol problems: systematic review of prospective cohort studies. J Epidemiol Community Health 2013; 68: 268–74. 24249000 10.1136/jech-2013-203402 PMC4158030

[54] McCambridge J. A case study of publication bias in an influential series of reviews of drug education. Drug Alcohol Rev 2007; 26: 463–8. 17701508 10.1080/09595230701494366

[55] Wagenaar A. C. , Salois M. J. , Komro K. A. Effects of beverage alcohol price and tax levels on drinking: a meta‐analysis of 1003 estimates from 112 studies. Addiction 2009; 104: 179–90. 19149811 10.1111/j.1360-0443.2008.02438.x

[56] Popova S. , Giesbrecht N. , Bekmuradov D. , Patra J. Hours and days of sale and density of alcohol outlets: impacts on alcohol consumption and damage: a systematic review. Alcohol Alcohol 2009; 44: 500–16. 19734159 10.1093/alcalc/agp054

[57] Koutakis N. , Stattin H. , Kerr M. Reducing youth alcohol drinking through a parent‐targeted intervention: the Örebro Prevention Program. Addiction 2008; 103: 1629–37. 18821873 10.1111/j.1360-0443.2008.02326.x

[58] Koning I. M. , Vollebergh W. A. , Smit F. , Verdurmen J. E. , Van Den Eijnden R. J. , Ter Bogt T. F. *et al.* Preventing heavy alcohol use in adolescents (PAS): cluster randomized trial of a parent and student intervention offered separately and simultaneously. Addiction 2009; 104: 1669–78. 21265908 10.1111/j.1360-0443.2009.02677.x

